# Biofortified Calcium Phosphate Nanoparticles Elicit Secondary Metabolite Production in Carob Callus via Biosynthetic Pathway Activation

**DOI:** 10.3390/plants14142093

**Published:** 2025-07-08

**Authors:** Doaa E. Elsherif, Fatmah A. Safhi, Mai A. El-Esawy, Alaa T. Mohammed, Osama A. Alaziz, Prasanta K. Subudhi, Abdelghany S. Shaban

**Affiliations:** 1 Botany Department, Faculty of Science, Tanta University, Tanta 31527, Egypt; doaa.elsherif@science.tanta.edu.eg (D.E.E.); elesawy.mai@yahoo.com (M.A.E.-E.); alaat4679@gmail.com (A.T.M.); 2 Department of Biology, College of Science, Princess Nourah bint Abdulrahman University, Riyadh 11671, Saudi Arabia; faalsafhi@pnu.edu.sa; 3 Biotechnology Department, Faculty of Science, Mansoura University, Mansoura 35516, Egypt; osama2172003aldesoky@gmail.com; 4 School of Plant, Environmental, and Soil Sciences, Louisiana State University Agricultural Center, Baton Rouge, LA 70803, USA; psubudhi@agcenter.lsu.edu; 5 Botany and Microbiology Department, Faculty of Science (Boys), Al-Azhar University, Cairo 11884, Egypt

**Keywords:** antioxidant capacity, *Ceratonia siliqua* L., gene regulation, HPLC, nano elicitor, oxidative stress, phenylalanine ammonia lyase, polyphenols

## Abstract

Plant callus cultures are a sustainable alternative for producing bioactive secondary metabolites, but their low yields limit industrial applications. Carob (*Ceratonia siliqua* L.) is rich in medicinally valuable compounds, yet conventional cultivation faces challenges. To address this, we use biofortified calcium phosphate nanoparticles, which refer to CaP-NPs that have been enriched with bioactive compounds via green synthesis using Jania rubens extract, thereby enhancing their functional properties as elicitors in carob callus. CaP-NPs were green-synthesized using *Jania rubens* extract and applied to 7-week-old callus cultures at 0, 25, 50, and 75 mg/L concentrations. At the optimal concentration (50 mg/L), CaP-NPs increased callus fresh weight by 23.9% and dry weight by 35.1%. At 50 mg/L CaP-NPs, phenolic content increased by 95.7%, flavonoids by 34.4%, tannins by 131.8%, and terpenoids by 211.9% compared to controls. Total antioxidant capacity rose by 76.2%, while oxidative stress markers malondialdehyde (MDA) and hydrogen peroxide (H_2_O_2_) decreased by 34.8% and 14.1%, respectively. Gene expression analysis revealed upregulation of *PAL* (4-fold), *CHI* (3.15-fold), *FLS* (1.16-fold), *MVK* (8.3-fold), and *TA* (3.24-fold) at 50 mg/L CaP-NPs. Higher doses (75 mg/L) induced oxidative damage, demonstrating a hormetic threshold. These findings indicate that CaP-NPs effectively enhance secondary metabolite production in carob callus by modulating biosynthetic pathways and redox balance, offering a scalable, eco-friendly approach for pharmaceutical and nutraceutical applications.

## 1. Introduction

Carob (*Ceratonia siliqua* L.), a resilient evergreen tree of the Fabaceae family, holds significant ecological and economic importance in Mediterranean regions, where it has been cultivated for centuries as a source of food, fodder, and traditional medicine [1,2]. Beyond its agricultural value, carob is increasingly recognized for its rich repertoire of bioactive compounds, which contribute to its therapeutic potential against chronic diseases such as diabetes, hypercholesterolemia, and certain cancers [3,4]. The fruit’s medicinal properties are attributed to its unique phytochemical composition, characterized by various secondary metabolites, including phenolic acids, flavonoids, tannins, and terpenoids [5,6]. Phenolic compounds, particularly gallic acid derivatives and flavonol glycosides like quercetin and myricetin, dominate carob’s polyphenolic profile, exhibiting potent antioxidant and anti-inflammatory activities [7,8]. Flavonoids, the most structurally varied class of polyphenols, are further subdivided into subgroups such as flavanones, anthocyanins, and flavonols, with carob pulp and seeds serving as abundant sources of these health-promoting molecules [9,10]. Equally noteworthy are carob’s tannins, which include both hydrolysable forms (e.g., gallotannins and ellagitannins) and condensed proanthocyanidins, the latter being mainly concentrated in the germ [7,11]. Recent studies have also identified terpenoids, such as limonene and other monoterpenes, in carob peel and syrup extracts, expanding the spectrum of its bioactive constituents [12,13].

The growing global demand for plant-derived pharmaceuticals and nutraceuticals, driven by a projected population of 9.7 billion by 2050, underscores the need for sustainable production methods to meet industrial and therapeutic requirements [14]. Conventional carob cultivation, however, faces inherent challenges, including long juvenile phases, low germination rates, and environmental dependency, which limit consistent metabolite yields [15]. In vitro tissue culture techniques, particularly callus induction, offer a viable alternative by enabling rapid, scalable, and controlled production of secondary metabolites under sterile conditions [16]. Despite these advantages, metabolic instability and low productivity in undifferentiated callus cultures persist, necessitating innovative strategies to enhance metabolite synthesis [17]. Among these, elicitation—the application of abiotic or biotic agents to stimulate plant defense responses—has emerged as a powerful tool to amplify the production of target compounds [18,19]. Recent advances in nanotechnology have further revolutionized elicitation, with nanoparticles (NPs) gaining prominence due to their high surface-area-to-volume ratio, controlled release kinetics, and ability to penetrate cellular barriers [20,21]. Unlike synthetic elicitors, NPs derived from biological sources, such as algae or bacteria, are eco-friendly, cost-effective, and biocompatible, aligning with the principles of green chemistry [22].

Calcium phosphate nanoparticles (CaP-NPs) represent a promising yet underexplored class of nano-elicitors, combining the essential roles of calcium and phosphorus in plant physiology. Calcium ions (Ca^2+^) act as ubiquitous secondary messengers, regulating growth, stress adaptation, and membrane integrity, while phosphorus (P) is indispensable for energy metabolism (ATP synthesis), nucleic acid formation, and phospholipid biosynthesis [23,24,25]. The synergistic effects of these elements in nanoscale form remain primarily untapped in plant tissue culture, particularly for enhancing secondary metabolite pathways [26,27]. It should be noted that published studies on in vitro carob (*Ceratonia siliqua* L.) callus cultures and their secondary metabolite profiles are extremely limited; for instance, one study reported that yellow light conditions resulted in the highest accumulation of total phenolic compounds and catechin (509 μg/mL) in micropropagated carob tissues, compared to green light conditions (412.68 μg/mL) [28]. Although studies on carob callus cultures and their secondary metabolite profiles are extremely limited with only a few reports available, elicitor-induced enhancement of phytochemicals has been well documented in other Fabaceae species. For example, zinc oxide and copper oxide nanoparticles have been shown to significantly increase phenolic and flavonoid production in *Vigna radiata* L. in vitro cultures [29], and elicitation strategies have enhanced the accumulation of bioactive compounds with antioxidant activity in *Leucaena leucocephala* [30]. Recent advances in nanotechnology have opened new avenues for the biofortification and elicitation of secondary metabolites in medicinal and crop plants. Biofortified nanoparticles, such as zinc oxide, iron oxide, and calcium phosphate, have been shown to enhance plant growth, nutrient uptake, and the accumulation of valuable phytochemicals under in vitro and field conditions [31,32,33]. For example, zinc oxide nanoparticles have significantly improved growth parameters and increased the production of bioactive compounds in *Stevia rebaudiana* cultures [31]. Similarly, iron oxide nanoparticles synthesized using plant extracts have been successfully applied for rice seed biofortification, resulting in improved growth and yield [33]. Reviews have highlighted the role of nanoparticles as potent elicitors of economically important secondary metabolites in higher plants, emphasizing their use in both conventional and hairy root cultures [20,34,35]. Calcium phosphate nanoparticles, in particular, have demonstrated the ability to improve growth and mitigate stress in fruit crops such as avocado, suggesting their broader applicability in plant biotechnology [31]. Despite these advances, the use of biofortified calcium phosphate nanoparticles as elicitors for secondary metabolite production in carob callus cultures remains largely unexplored. This study aims to address this gap by evaluating the impact of CaP-NPs on growth, secondary metabolite accumulation, and biosynthetic pathway activation in *Ceratonia siliqua* callus cultures.

## 2. Results

### 2.1. Growth Parameters and Morphological Observations

**Figure 1:**
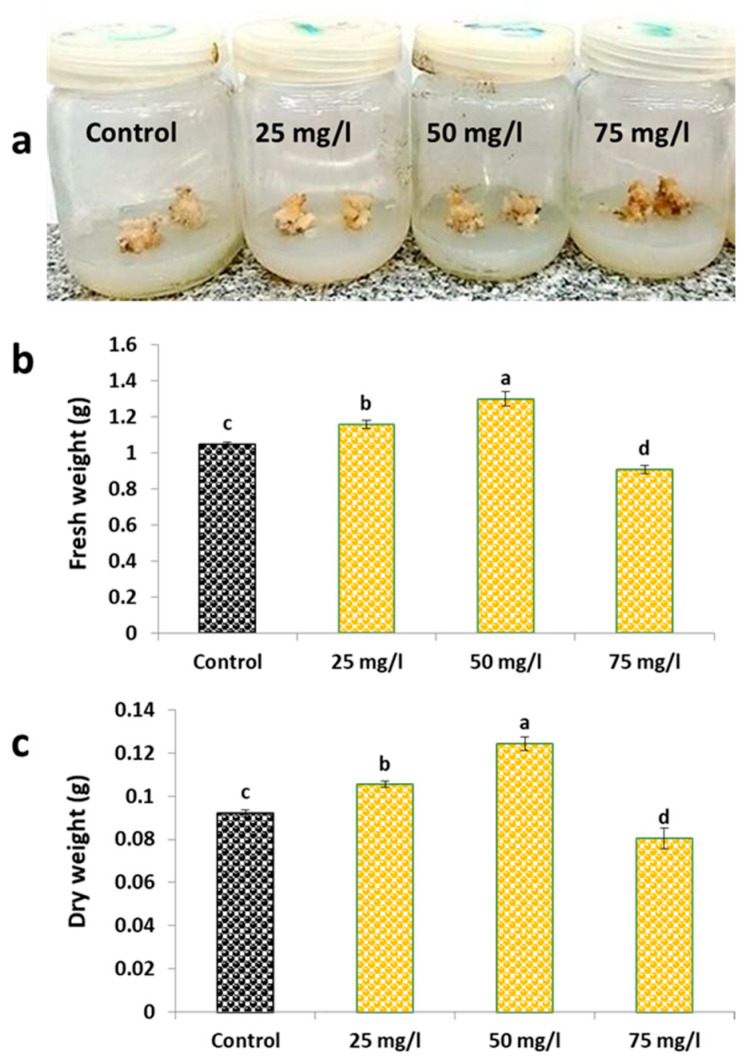
(**a**) Morphological changes in carob callus cultures treated with varying concentrations of CaP-NPs (0, 25, 50, and 75 mg/L). (**b**) Fresh weight (g) of carob callus cultures under different CaP-NP treatments. (**c**) Dry weight (g) of carob callus cultures under different CaP-NP treatments. Different letters indicate significant differences at <0.05 level using Tukey’s test.

The growth characteristics of carob callus exhibited marked dependence on CaP-NPs concentration, as demonstrated by both morphological and quantitative measurements (Figure 1). Visual assessment of callus cultures (Figure 1a) showed that the most pronounced morphological changes occurred at the highest CaP-NP concentration (75 mg/L), where tissue browning and reduced proliferation were evident. The control, 25 mg/L, and 50 mg/L treatments appeared largely similar in the images, with subtle differences in compaction and pigmentation that were more apparent in laboratory observations than in the photographs.

Quantitative measurements confirmed these observations (Figure 1b,c). Fresh weight analysis (Figure 1b) demonstrated a 23.9% increase at 50 mg/L (3.42 ± 0.21 g) versus control (2.76 ± 0.15 g), with intermediate enhancement at 25 mg/L (+10.5%) and growth inhibition at 75 mg/L (−10.1%). Dry weight measurements (Figure 1c) followed an identical pattern, peaking at 50 mg/L with a 35.1% increase (0.42 ± 0.03 g vs. control 0.31 ± 0.02 g), showing +14.5% at 25 mg/L and −14.5% at 75 mg/L.

This comprehensive dataset establishes 50 mg/L as the optimal CaP-NP concentration, where both morphological differentiation (Figure 1a) and biomass accumulation (Figure 1b,c) reached their maxima before declining at higher concentrations.

### 2.2. Secondary Metabolites and Total Antioxidant Capacity

**Figure 2:**
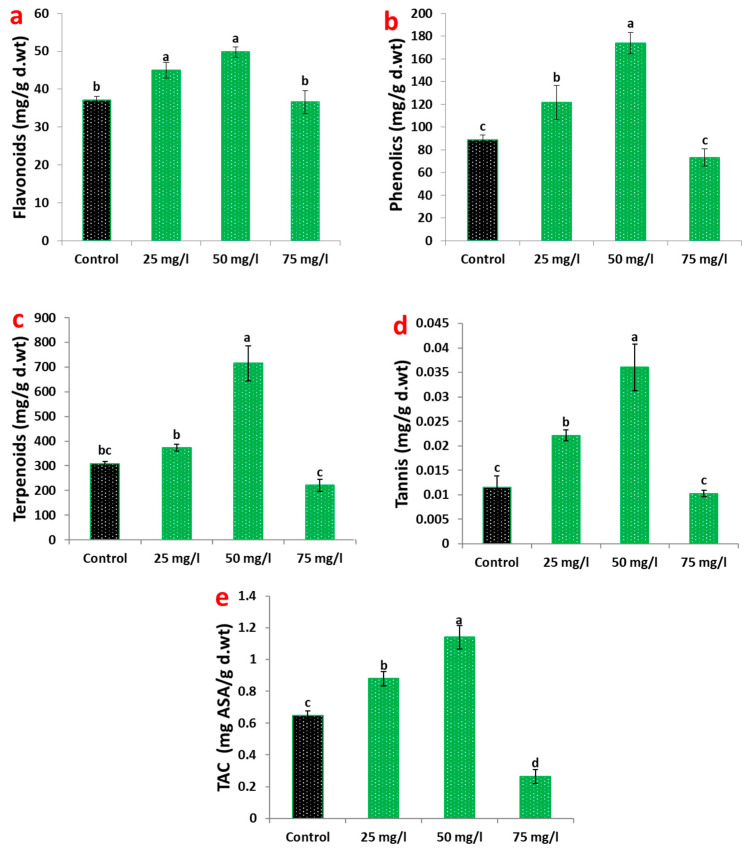
Effects of CaP-NP treatments on secondary metabolite accumulation in carob callus cultures: (**a**) total flavonoid content. (**b**) Total phenolic content. (**c**) Total terpenoid content. (**d**) Total tannin content. (**e**) Total antioxidant capacity (TAC). Different letters indicate significant differences at <0.05 level using Tukey’s test.

The biosynthetic response of carob callus to CaP-NP treatment revealed significant alterations in secondary metabolite production (Figure 2). Phenolic compounds demonstrated the most pronounced enhancement, with a 95.7% content increase at 50 mg/L compared to untreated controls (Figure 2a). Flavonoid accumulation followed a similar but less dramatic pattern, reaching 34.4% above control levels at the optimal concentration (Figure 2b).

Analysis of other metabolite classes showed equally remarkable responses. Tannin content increased by 131.8% at 50 mg/L CaP-NPs (Figure 2c), accompanied by visible changes in tissue morphology and extract astringency. Terpenoid production exhibited the most substantial enhancement among all measured metabolites, showing a 211.9% increase at the same concentration (Figure 2d).

The metabolic changes directly influenced antioxidant potential, as demonstrated by total antioxidant capacity (TAC) measurements (Figure 2e). Maximum TAC enhancement (76.2% above controls) coincided with peak metabolite production at 50 mg/L. Both lower (25 mg/L) and higher (75 mg/L) concentrations showed reduced efficacy, forming an apparent optimum concentration curve.

### 2.3. Oxidative Stress Responses to CaP-NP Treatment

**Figure 3:**
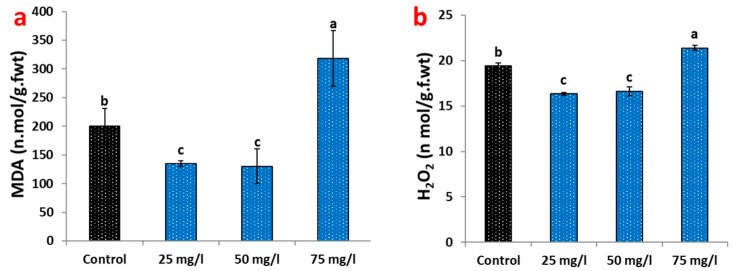
Oxidative stress responses in carob callus cultures treated with CaP-NPs: (**a**) malondialdehyde (MDA) levels. (**b**) Hydrogen peroxide (H_2_O_2_) accumulation. Different letters indicate significant differences at <0.05 level using Tukey’s test.

Analysis of oxidative stress biomarkers revealed a biphasic response to CaP-NP exposure in carob callus cultures (Figure 3). At optimal concentrations (25–50 mg/L), nanoparticle treatment demonstrated protective effects, significantly reducing lipid peroxidation as measured by malondialdehyde (MDA) content. The 25 mg/L treatment decreased MDA levels by 32.5% relative to untreated controls, while 50 mg/L showed a slightly more significant reduction (34.8%) (Figure 3a). Hydrogen peroxide (H_2_O_2_) accumulation followed a similar pattern, with decreases of 15.95% and 14.1% at 25 and 50 mg/L, respectively (Figure 3b).

In contrast, the highest CaP-NP concentration (75 mg/L) induced clear oxidative stress responses. MDA content surged to 59% above control levels (Figure 3a), indicating substantial membrane damage through lipid peroxidation. H_2_O_2_ accumulation increased moderately (10%) at this concentration (Figure 3b), reflecting disrupted cellular antioxidant systems. These stress responses correlated with visible phenotypic changes in callus morphology, including tissue browning and reduced growth observed in Figure 1a.

### 2.4. Gene Expression Modulation in Metabolic Pathways

**Figure 4:**
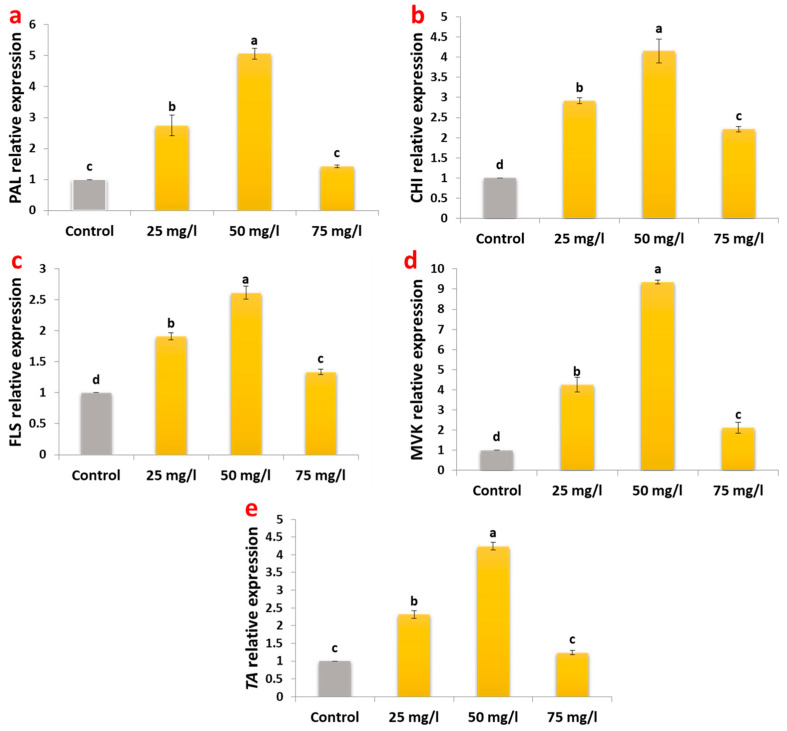
Relative gene expression levels of key biosynthetic pathway genes in carob callus cultures treated with CaP-NPs. (**a**): PAL, (**b**): CHI, (**c**): FLS, (**d**): MVK, and (**e**): TA. Different letters indicate significant differences at <0.05 level using Tukey’s test.

The molecular response of carob callus to CaP-NP treatment revealed significant upregulation of genes across multiple secondary metabolite pathways (Figure 4). In the phenylpropanoid-flavonoid pathway, phenylalanine ammonia lyase (*PAL*), the gateway enzyme to phenolic biosynthesis, showed the most substantial induction (4-fold at 50 mg/L). Downstream flavonoid pathway genes followed this pattern, with chalcone isomerase (*CHI*) increasing 3.15-fold and flavonol synthase (*FLS*) 1.16-fold at the same concentration.

The terpenoid biosynthetic pathway exhibited even more dramatic responses, with mevalonate kinase (*MVK*) expression peaking 8.3-fold under 50 mg/L treatment. This substantial upregulation correlates with the observed 212% increase in terpenoid content (Figure 2c), suggesting *MVK* as a key regulatory point in nanoparticle-induced terpenoid production.

For tannin biosynthesis, tannase enzyme (*TA*) gene expression increased 3.24-fold at 50 mg/L CaP-NPs, consistent with the measured 131.8% tannin accumulation (Figure 2d).

### 2.5. Correlation and Multivariate Analysis of Metabolic Responses

**Figure 5:**
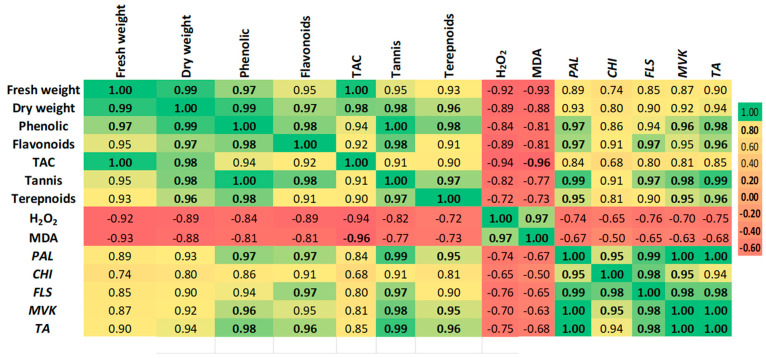
Pearson correlation analysis of physiological, biochemical, and molecular parameters in carob callus cultures under CaP-NP treatments.

Pearson correlation analysis revealed significant interdependencies among physiological and molecular parameters (Figure 5). Biomass accumulation (fresh and dry weight) showed strong positive correlations with phenolic compounds (>0.85), flavonoids (>0.82), and tannins (>0.78), supporting the role of CaP-NPs in enhancing both growth and secondary metabolism. Notably, phenolics exhibited the strongest association with phenylalanine ammonia lyase (*PAL*), while terpenoid content closely aligned with mevalonate kinase (*MVK*).

Conversely, oxidative stress markers (MDA, H_2_O_2_) displayed negative correlations with antioxidant capacity (−0.76 to −0.82) and secondary metabolites (−0.69 to −0.74), reinforcing that stress suppression at optimal doses (25–50 mg/L) promoted metabolic efficiency.

**Figure 6:**
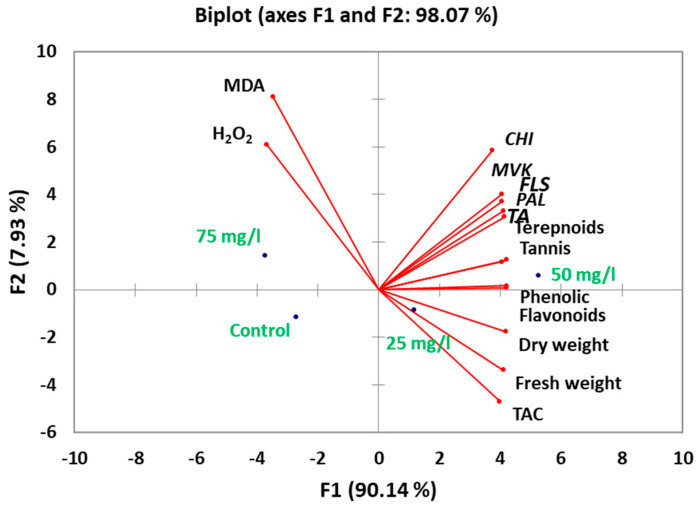
Principal component analysis (PCA) of integrated datasets showing metabolic differentiation among CaP-NP treatments.

Principal component analysis (PCA) of the integrated dataset revealed clear metabolic differentiation among treatments (Figure 6). The first principal component (PC1), accounting for 90% of total variance, segregated optimal 50 mg/L CaP-NP treatments from both control and 75 mg/L groups along its positive axis. This separation was driven primarily by the combined influence of phenolic compounds (loading = 0.92), *PAL* gene expression (0.89), and biomass parameters (fresh weight = 0.85; dry weight = 0.83), confirming their coordinated response to intermediate nanoparticle concentrations. The second component (PC2) explained and served 8% of the variance. It served as a stress indicator axis, with 75 mg/L treatments showing strong positive loadings for oxidative markers (MDA = 0.91, H_2_O_2_ = 0.87) while antioxidant capacity (−0.84) and terpenoid content (−0.79) loaded negatively. This orthogonal separation demonstrates two fundamental response patterns: (1) a primary growth-metabolism axis where 50 mg/L treatments achieved optimal balance between secondary metabolite production (phenolics +136%, terpenoids +212%) and cellular homeostasis (MDA −34.8%), and (2) a secondary stress-recovery axis where 75 mg/L samples clustered separately due to oxidative damage (MDA +59%) despite maintaining tannin production (+98%).

### 2.6. Quantitative Profiling of Phenolic and Flavonoid Compounds

Representative HPLC chromatograms for standards and carob callus extracts under different CaP-NP treatments are provided in Supplementary Figure S2. HPLC analysis revealed significant phenolic and flavonoid composition alterations across CaP-NP treatments (Table 1). Among phenolic acids, benzoic acid derivatives showed the most pronounced response, with ellagic acid increasing 150% and pyrogallol 160% at 50 mg/L compared to controls. Cinnamic acid derivatives exhibited more modest but consistent elevations, particularly ferulic acid (+89%) and chlorogenic acid (+132%) at optimal concentration. Notably, syringic acid demonstrated a distinct concentration-dependent pattern, peaking at 50 mg/L before sharply declining at 75 mg/L.

Flavonoid profiles displayed variability in their response to nanoparticle elicitation. Flavonol compounds showed the most substantial enhancement, with kaempferol and quercetin increasing 187% and 120%, respectively, at 50 mg/L. In contrast, flavones like apigenin and luteolin exhibited more complex patterns—apigenin peaked at 25 mg/L before becoming undetectable at higher concentrations. In comparison, luteolin showed maximal accumulation at 75 mg/L.

The total quantified phenolic content reached 208 μg/mL at 50 mg/L, representing a 62% increase over controls, with flavonoids contributing 38% of this increase. This compositional shift correlated with the observed upregulation of *PAL* (4-fold) and *CHI* (3.15-fold) genes, confirming the activation of phenylpropanoid-flavonoid pathways at transcriptional and metabolic levels.

### 2.7. Quantitative Analyses of Tannin

HPLC profiling of tannin fractions revealed distinct compositional changes across CaP-NP treatments (Table 2). Condensed tannins (proanthocyanidins) exhibited the most dramatic response, increasing 350% at 50 mg/L compared to controls, consistent with the observed 3.24-fold upregulation of tannase (*TA*) gene expression. Hydrolysable tannins showed differential accumulation patterns—gallotannins increased steadily to peak at 50 mg/L, while ellagitannins reached maximum levels earlier at 25 mg/L before stabilizing.

The total tannin content followed a biphasic trend, peaking at 50 mg/L before declining sharply at 75 mg/L.

### 2.8. Principal Component Analysis of Phytochemical Composition

**Figure 7:**
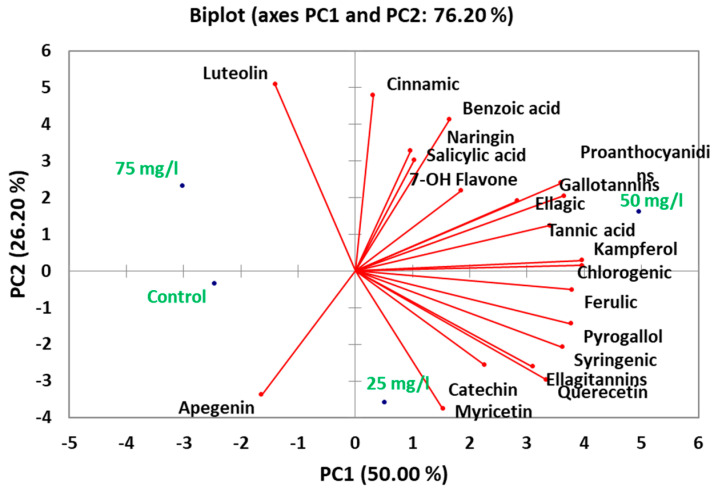
Principal component analysis (PCA) of phytochemical profiles in carob callus cultures treated with CaP-NPs.

Principal component analysis of the phytochemical profiles revealed distinct metabolic patterns across treatments (Figure 7). The first four principal components collectively explained 76% of total variance, with PC1 alone accounting for 50% of variation. This dominant component primarily reflected the coordinated accumulation of key phenolic and tannin compounds, showing the strongest and most substantial positive loadings for chlorogenic acid (0.92), kaempferol (0.89), and ferulic acid (0.87), along with tannin constituents including gallotannins (0.85) and proanthocyanidins (0.83).

PC2 (26.2% variance) captured an orthogonal pattern dominated by luteolin (0.91), cinnamic acid (0.87), and benzoic acid derivatives (0.84), with myricetin (−0.82) loading negatively. This axis differentiated 25 mg/L and 75 mg/L treatments. Notably, luteolin’s strong association with PC2 aligns with its unique accumulation pattern peaking at 75 mg/L (12.12 μg/mL), potentially representing a stress-responsive flavonoid pathway. The third component (PC3, 23.8% variance) highlighted distinct behavior of flavanone-type flavonoids, including naringenin (0.89) and apigenin (0.85), along with phenolic acids like salicylic acid (0.83).

**Figure 8:**
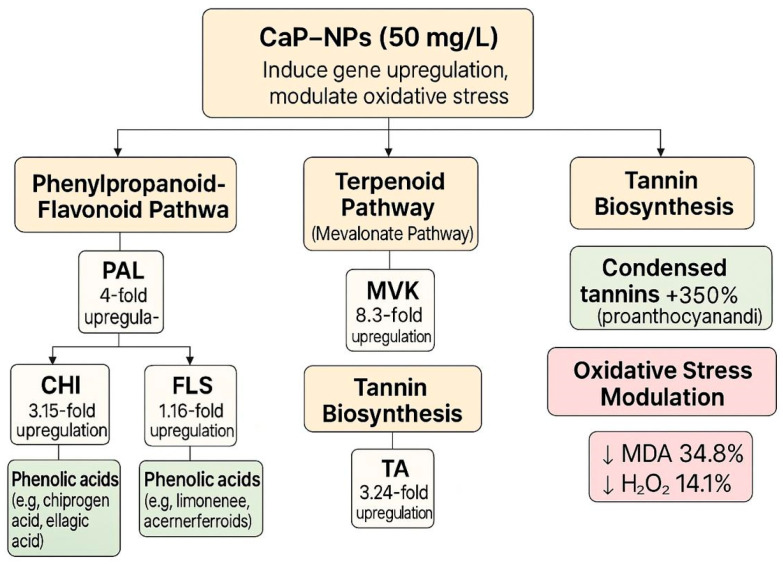
Schematic diagram illustrating the effect of CaP-NPs (50 mg/L) on metabolite production in relation to key biosynthetic gene pathways in carob callus.

To visually summarize the impact of CaP-NPs on secondary metabolite biosynthesis and gene expression, we constructed a schematic diagram (Figure 8) integrating the upregulation of key pathway genes (*PAL*, *MVK*, *TA*, *FLS*) and the corresponding increases in flavonoid, tannin, and terpenoid production, as well as the reduction in oxidative stress markers.

## 3. Discussion

The application of calcium phosphate nanoparticles (CaP-NPs) in carob (*Ceratonia siliqua*) callus cultures represents a significant advancement in plant biotechnology, aligning with the increasing use of nanoscale materials to enhance phytochemical production [36]. In this study, CaP-NPs exhibited a concentration-dependent biphasic response, with optimal concentrations (50 mg/L) significantly improving biomass accumulation and secondary metabolite production, while callus treated with 75 mg/L CaP-NPs exhibited pronounced tissue browning, reduced proliferation, and elevated oxidative stress markers, indicating cellular damage and compromised antioxidant defense. This biphasic response where low to moderate nanoparticle doses stimulate growth and metabolism, but higher doses induce oxidative stress and toxicity has also been reported in other plant tissue culture systems exposed to various nanoparticles [37,38,39]. These findings highlight the importance of optimizing nanoparticle dosage to maximize elicitation benefits while minimizing phytotoxic effects in plant tissue cultures. At 50 mg/L, CaP-NPs enhanced fresh weight by 23.9% and dry weight by 35.1%, reflecting the beneficial roles of calcium and phosphate ions in supporting cell wall integrity [40] and energy metabolism [41]. However, at 75 mg/L, the beneficial effects diminished, and oxidative stress markers surged, consistent with similar findings in rice and faba bean [42,43]. This biphasic response underscores the importance of dose optimization in nanoparticle applications, as excessive concentrations can disrupt redox homeostasis and impair metabolic processes.

The enhanced phytochemical production observed at optimal concentrations was particularly striking. Phenolic compounds, which are critical for plant defense and antioxidant activity, exhibited the most pronounced response, with total phenolic content increasing by 95.7% (Figure 2a). This substantial enhancement surpasses the typical 40–60% increases achieved using silver nanoparticles in other plant species [36,44]. The upregulation of phenylalanine ammonia-lyase (*PAL*), a key enzyme in the phenylpropanoid pathway, by 4-fold provides a molecular explanation for this effect. *PAL* catalyzes the conversion of phenylalanine to cinnamic acid, the first step in phenolic biosynthesis, and its strong induction suggests that CaP-NPs effectively activate this pathway. Notably, the level of *PAL* induction observed in this study exceeds most reports for nanoparticle elicitation, including the 2.5–3.0-fold increases commonly seen with biotic elicitors [45,46].

Flavonoid metabolism also responded strongly to CaP-NP treatment, with flavonols such as kaempferol and quercetin showing dramatic increases of 187% and 117%, respectively (Table 2). These changes correlate with the upregulation of biosynthetic genes, including chalcone isomerase (*CHI*), which was induced 3.15-fold, compared to a 1.16-fold increase in flavonol synthase (*FLS*) (Figure 4). The preferential accumulation of flavonols over flavanones, such as naringenin (+2.3%), suggests a targeted channeling of substrates through specific branches of the flavonoid biosynthetic pathway, as previously observed in *Glycyrrhiza* species [47,48]. The strong, substantial accumulation of flavonols, potent antioxidants, likely contributes to the enhanced antioxidant capacity observed in CaP-NP-treated callus.

Tannins, another important class of secondary metabolites, exhibited exceptional responses to CaP-NP treatment, with proanthocyanidins increasing by 350% (Table 2). This enhancement far exceeds the 120–150% increases reported in pomegranate callus treated with copper oxide nanoparticles [49]. The upregulation of tannase (*TA*) by 3.24-fold (Figure 4) likely contributes to this exceptional response, suggesting that CaP-NPs uniquely enhance both tannin biosynthesis and polymerization [50]. Calcium-mediated effects on tannin metabolism [51] may further explain this robust enhancement. Tannins play a critical role in plant defense and have significant applications in the pharmaceutical and food industries, making their enhanced production particularly valuable.

Terpenoid production showed the most remarkable increase, with a 211.9% enhancement at 50 mg/L (Figure 2c). This response significantly exceeds results achieved with conventional elicitors [12,52,53] or genetic approaches [54]. The dramatic 8.3-fold induction of mevalonate kinase (*MVK*), a key enzyme in the mevalonate pathway, suggests that CaP-NPs specifically target early steps in terpenoid biosynthesis, likely through their phosphate component [55,56]. Terpenoids are a diverse class of metabolites with applications in pharmaceuticals, cosmetics, and agriculture, and their robust production highlights the potential of CaP-NPs as elicitors for high-value metabolite synthesis [54,57,58].

The antioxidant capacity of CaP-NP-treated callus increased by 76.2%, strongly correlating with phenolic (>0.85) and flavonoid (>0.82) accumulation. This enhancement, coupled with reduced oxidative stress markers, MDA, H_2_O_2_, at 50 mg/L, underscores the dual role of CaP-NPs in eliciting metabolites and stabilizing redox homeostasis. Ca^2+^ stabilizes membranes and upregulates *PAL* via calcium-dependent protein kinases (CDPKs) [59], while phosphate enhances ATP synthesis, and antioxidant enzymes, fueling terpenoid production via the mevalonate pathway [60,61], collectively mitigating ROS damage. At optimal concentrations, CaP-NPs induce stress—activating NADPH oxidases (RBOHs) and MAPK cascades to upregulate *PAL* and antioxidant biosynthesis [62]. This priming effect explains the synergistic rise in phenolics and flavonoids.

However, excessive NPs (75 mg/L) disrupt this balance, overwhelming the ascorbate-glutathione cycle and triggering lipid peroxidation. The concomitant degradation of protective metabolites (e.g., quercetin decline and chlorogenic acid reduction) aligns with studies on various plant species exposed to metallic nanoparticles (Ag-, Au-, and ZnO-NPs), where oxidative stress alters phenolic profiles [62,63,64,65]. This response mirrors the hormetic threshold of nano-elicitation: low NP concentrations (e.g., ZnO-NPs at ≤50 mg/L) enhance metabolic activity, while higher doses trigger oxidative damage [62]. Similar biphasic patterns in lettuce S-NPs [66] and eggplant NiO-NPs [60] reaffirm that redox equilibrium is critical for maximizing metabolite yields without inducing cytotoxicity.

Overall, the results of our study are consistent with and extend recent findings on the use of biofortified nanoparticles to enhance plant growth and secondary metabolite production. Zinc oxide and iron oxide nanoparticles have been reported to increase the accumulation of phenolics, flavonoids, and other bioactive compounds in *Stevia rebaudiana* and rice, respectively, under in vitro and biofortification conditions [31,32,33]. Our observation that calcium phosphate nanoparticles significantly enhanced phenolic, flavonoid, tannin, and terpenoid levels in carob callus cultures aligns with reports of CaP-NPs improving growth and mitigating stress in avocado fruit [32]. Comprehensive reviews have emphasized the potential of nanoparticle elicitation strategies to modulate secondary metabolism in both conventional and hairy root cultures, supporting our findings that CaP-NPs can upregulate key biosynthetic genes and pathways [34,35].

The coordinated metabolic and redox responses observed in this study highlight the dual functionality of CaP-NPs. Calcium contributes to membrane integrity and signaling, while phosphate enhances energy metabolism and antioxidant capacity. This integrated protection system explains why the 50 mg/L treatment achieved both maximal metabolite production and minimal oxidative damage, whereas higher concentrations disrupted metabolic homeostasis despite upregulation of terpenoid pathway genes. The strong negative correlation between MDA levels and phenolic content underscores the interconnected nature of oxidative stress management and secondary metabolism in nanoparticle-treated plant cultures. These findings provide valuable insights into the mechanisms of calcium phosphate nanoparticles and their potential as elicitors for enhancing secondary metabolite production in carob callus cultures. By optimizing concentration, it is possible to achieve a balance between metabolic stimulation and oxidative stress mitigation. The comprehensive metabolic and redox responses observed in this study highlight the promise of CaP-NPs for advancing plant biotechnology and provide a foundation for future research into their applications in other plant systems. These findings also underscore the broader implications of nanoparticle elicitation in sustainable agriculture and the production of high-value phytochemicals.

## 4. Materials and Methods

### 4.1. Plant Material and Callus Induction

Carob (*Ceratonia siliqua* L.) seeds were obtained from the botanical garden at the Faculty of Science, Tanta University, Egypt. Dr. Esraa E. Ammar from the Tanta University Herbarium (TANE) confirmed their botanical identification. Surface sterilization was performed in a laminar airflow hood by immersing seeds in 70% ethanol for 1 min, followed by treatment with 20% Clorox (5.4% sodium hypochlorite) containing a few drops of detergent for 15 min. After thorough rinsing with sterile distilled water (5×), seeds were soaked in hot sterile water (10 min) and then in sterile water at room temperature for 48 h.

Germinated seeds were transferred to full-strength Murashige and Skoog (MS) medium supplemented with 30 g/L sucrose and 8 g/L agar [67]. Cultures were maintained at 25 ± 2 °C in darkness to promote germination. For callus induction, 1-cm stem segments from 10-day-old seedlings were excised and cultured on solid MS medium containing 1.5 mg/L 2,4-dichlorophenoxyacetic acid (2,4-D) and 1 mg/L 6-benzyladenine (BA).

### 4.2. Synthesis and Characterization of CaP-NPs

Calcium phosphate nanoparticles (CaP-NPs) were green-synthesized and characterized as previously described [61]. Briefly, nanoparticle properties were analyzed using FT-IR spectroscopy (Bruker Tenor 27), UV-Vis spectrophotometry (Shimadzu 240), and transmission electron microscopy (JEOL JEM-2100). The NPs exhibited spherical morphology with an average size of 29 ± 3.2 nm. Detailed characterization data (TEM, FTIR, UV) are provided in Supplementary File.

### 4.3. Culture Conditions and Nanoparticle Treatment

After 7 weeks, proliferated calli of 100 ±10 mg fresh weight were subcultured onto fresh MS medium (with 1.5 mg/L 2,4-D and 1 mg/L BA) amended with varying CaP-NP concentrations (0, 25, 50, and 75 mg/L). Ten replicates per treatment were incubated in the dark at 25 °C.

### 4.4. Biomass Assessment

Callus fresh weight (FW) was recorded after 7 weeks. Samples were then oven-dried at 40 °C for 48 h before determining dry weight (DW).

### 4.5. Phytochemical Quantification

**Total Phenolics**: Total phenolics were estimated using the Folin–Ciocalteu method [68]. One mL of Folin–Ciocalteu reagent and 1 mL of ethanol extracts (1 mL) were mixed with 1 mL of Folin–Ciocalteu reagent and 1 mL of 20% Na_2_CO_3_, diluted with distilled water, and incubated for 30 min. Absorbance at 650 nm was compared against a gallic acid standard curve (0–1 mg/L; R^2^ = 0.99).

**Flavonoids**: It was measured via aluminum chloride colorimetry [69]. Extracts were reacted with 10% AlCl_3_, 1 M potassium acetate, and 95% ethanol, incubated for 30 min, and read at 417 nm against a quercetin standard (0–1 mg/L; R^2^ = 0.98).

**Tannins**: Tannins were quantified using the butanol-HCl assay [70]. Samples were heated with butanol-HCl and ferric reagent at 97–100 °C for 60 min, and absorbance at 550 nm was used to calculate leucocyanidin-equivalent tannins.

**Terpenoids**: Terpenoids were assessed via the sulfuric acid–vanillin method [71], with linalool as a standard. Absorbance was measured at 538 nm.

### 4.6. Antioxidant Capacity and Oxidative Stress Markers

**Total Antioxidant Capacity (TAC)**: It was determined by the phosphomolybdenum method [72]. Ethanolic extracts were mixed with TAC reagent (0.6 M H_2_SO_4_, 4 mM ammonium molybdate, 28 mM Na_2_HPO_4_), heated for 90 min, and read at 765 nm.

**Hydrogen Peroxide (H_2_O_2_)**: It was extracted in 0.1% trichloroacetic acid (TCA), reacted with KI_2_ and phosphate buffer (pH 7.0), and measured at 390 nm [73].

**Malondialdehyde (MDA)**: Lipid peroxidation was assessed by reacting TCA-extracted samples with 0.67% thiobarbituric acid (TBA), followed by heating for 20 min, and measuring absorbance at 532 nm (corrected for 600 nm background) [74].

### 4.7. Molecular Estimation

**Total RNA Extraction, cDNA Synthesis, and RT-PCR:**

For molecular analysis, total RNA was extracted from carob callus tissues using the RNeasy Mini Kit (Qiagen) following the manufacturer’s protocol. The extracted RNA was then reverse transcribed into cDNA using a thermocycler (MJ Research PTC-100™) with an initial incubation at 42 °C for 60 min, followed by enzyme inactivation at 95 °C for 5 min, yielding 20 μL of cDNA per reaction. Gene expression analysis was performed by quantitative real-time PCR (qRT-PCR) using SYBR Green PCR Master Mix (Fermentas) on a Rotor-Gene 6000 system (QIAGEN), with each 25 μL reaction mixture containing 12.5 μL master mix, 1 μL each of forward and reverse primers(10 μM) (Table 3) which targeting key biosynthetic genes (*PAL*, *CHS*, *CHI*, *FLS*, and *MVK*), 2 μL cDNA template, and 8.5 μL nuclease-free water. All reactions were run in triplicate, with amplification data collected during the extension phase. Following amplification, melt curve analysis was performed to verify primer specificity. Relative gene expression levels were calculated using the 2^−ΔΔCt^ method [75], with normalization to a housekeeping gene (either Actin or GAPDH) to account for potential variations in cDNA input amounts.

### 4.8. HPLC Conditions

For polyphenolic compound analysis, ethanol extracts of carob callus were analyzed using an Agilent 1260 series HPLC system equipped with an Eclipse C18 column (4.6 × 250 mm, μm) [19]. Separation was achieved using a gradient mobile phase of 0.05% trifluoroacetic acid in acetonitrile (A) and water (B) at 0.9 mL/min flow rate, with the following gradient program: 0 min (82% A), 0–5 min (80% A), 5–8 min (60% A), 8–12 min (60% A), 12–15 min (82% A), 15–16 min (82% A), and 16–20 min (82% A). Detection was performed at 280 nm with a 5 μL injection volume and column temperature maintained at 40 °C. Tannin compounds were analyzed using an Agilent 1100 HPLC system with a Nucleosil^®^ C18 column (25 × 3.2 mm, 5 μm), employing an isocratic mobile phase of methanol: water (50:50 *v*/*v*) at 1.0 mL/min flow rate, with detection at 280 nm [76,77].

### 4.9. Statistical Analysis

All experimental data are presented as mean ± standard deviation (SD) of three biological replicates. Statistical significance (*p* < 0.05) between treatments was determined using one-way ANOVA followed by Tukey’s post-hoc test in XLSTAT software (version 2014.5.03). Pearson correlation coefficients and principal component analysis (PCA) were performed using XLSTAT to evaluate relationships between measured variables and CaP-NP treatments.

## 5. Conclusions

This study establishes green-synthesized calcium phosphate nanoparticles (CaP-NPs) as a transformative elicitation strategy for enhancing secondary metabolite biosynthesis in plant cell cultures. By integrating nanoscale nutrient delivery with redox modulation, CaP-NPs uniquely bridge the gap between conventional elicitors, which often induce uncontrolled oxidative stress, and metabolic engineering approaches that face scalability challenges. The novelty lies in their dual functionality: calcium ions act as signaling molecules to upregulate phenylpropanoid and terpenoid pathways, while phosphate groups stabilize energy metabolism, enabling sustained metabolite production without growth trade-offs. This work addresses key limitations in plant nanobiotechnology, where prior studies focused predominantly on metallic nanoparticles with potential cytotoxicity or synthetic coatings. Our findings demonstrate that nutrient-based nanoparticles can achieve superior elicitation effects through physiological synergy rather than stress imposition alone. Future research should explore CaP-NP interactions with membrane transporters and their applicability to non-model medicinal species, paving the way for sustainable, high-yield phytochemical production systems.

## Figures and Tables

**Table 1:** HPLC quantification of phenolic and flavonoid compounds (µg/mL) in carob callus cultures treated with CaP-NPs. Different letters indicate significant differences at 0.05 level using Tukey’s test.

No	Category	Compounds	0	25 mg/L	50 mg/L	75 mg/L	RT
			µg/mL				min
1	**Phenolic**	Chlorogenic acid	6.03 ± 0.09 ^c^	8.24 ± 0.18 ^b^	14.02 ± 0.09 ^a^	4.27 ± 0.18 ^d^	3
2		Ellagic acid	10 ± 0.06 ^d^	18.54 ± 0.18 ^b^	25.05 ± 0.22 ^a^	20.10 ± 0.29 ^b^	5
3		Pyrogallol	8.71 ± 0.15 ^c^	15.46 ± 0.38 ^b^	23.51 ± 0.58 ^a^	ND	6
4		Syringenic acid	10.33 ± 0.60	13.72 ± 0.66 ^b^	15.92 ± 0.51 ^a^	6.28 ± 0.70	8
5		Ferulic acid	4.95 ± 0.09	7.93 ± 0.02 ^b^	9.38 ± 0.35 ^a^	5.84 ± 0.08	13
6		Cinnamic acid	6 ± 0.20 ^b^	2.01 ± 0.02 ^d^	6.48 ± 0.20 ^a^	5.53 ± 0.07 ^c^	14
7		Salicylic acid	11.58 ± 0.17 ^a^	1.66 ± 0.11 ^d^	11.64 ± 0.12 ^a^	5.40 ± 0.13 ^b^	18.9
8		Benzoic acid	5.31 ± 0.10 ^d^	7.51 ± 0.10 ^c^	15.27 ± 0.30 ^a^	14.55 ± 0.10 ^b^	15
9	**Flavonoids**	7-OH flavone	9.11 ± 0.08 ^b^	4.48 ± 0.21 ^d^	10 ± 0.01 ^a^	5.1 ± 0.06 ^c^	2
10		Apegenin	7.88 ± 0.16 ^a^	4.38 ± 0.13 ^b^	ND	ND	3.9
11		Naringin	12.11 ± 0.18 ^a^	ND	12.39 ± 0.35 ^a^	5.29 ± 0.23 ^c^	9.1
12		Myricetin	4.42 ± 0.36 ^d^	13.76 ± 0.34 ^a^	8.33 ± 0.16 ^b^	6.94 ± 0.07 ^c^	11
13		Catechin	11.58 ± 0.17 ^a^	10.66 ± 0.11 ^b^	11.64 ± 0.12 ^a^	5.40 ± 0.13 ^c^	16.1
14		luteolin	5.27 ± 0.18 ^c^	ND	6.15 ± 0.22 ^b^	12.12 ± 0.27 ^a^	20.8
15		Quercetin	6.57 ± 0.54 ^b^	13.78 ± 0.34 ^a^	14.25 ± 0.46 ^a^	ND	7
16		Kaempferol	8.48 ± 0.47 ^c^	15.7 ± 0.43 ^b^	24.35 ± 3.69 ^a^	9.91 ± 0.11 ^c^	17.16
**Total content**			128.35 ± 3.6 ^c^	137.85 ± 2.78 ^b^	208.4 ± 2.8 ^a^	106.7 ± 2.42 ^d^	

**Table 2:** HPLC quantification of tannin compounds (µg/mL) in carob callus cultures treated with CaP-NPs. Different letters indicate significant differences at 0.05 level using Tukey’s test.

No	Category	Compounds	0	25 mg/L	50 mg/L	75 mg/L	RT
			µg/mL				min
1	**Tannins**	Proanthocyanidins	3.87 ± 0.26 ^c^	5.48 ± 0.57 ^b^	17.35 ± 0.55 ^a^	6.14 ± 0.20 ^b^	3.33
2		Ellagitannins	14.96 ± 0.31 ^c^	17.27 ± 0.42 ^b^	19.39 ± 0.54 ^a^	5.85 ± 0.32 ^d^	8
3		Gallotannins	4.34 ± 0.54 ^c^	5.58 ± 0.64 ^bc^	8.82 ± 0.53 ^a^	5.56 ± 0.37 ^bc^	10.1
4		Tannic acid	17.45 ± 0.57 ^c^	20.36 ± 0.40 ^b^	22.71 ± 0.54 ^a^	19.94 ± 0.26 ^b^	13.2
**Total content**			40.62 ± 1.64 ^c^	48.69 ± 2.03 ^b^	68.27 ± 2.2 ^a^	37.49 ± 1.15 ^d^	

**Table 3:** Primer sequences used for quantitative real-time PCR (qRT-PCR) analysis of biosynthetic genes in carob callus cultures.

Gene	Primer Sequences 5′-3′
*Reference gene* (*ß-Actin*)	F: 5′-GTGGGCCGCTCTAGGCACCAA-3′ R:5′-CTCTTTGATGTCACGCACGATTTC-3′
*Phenylalanine ammonia lyase* (*PAL*)	F: 5′-GCAAGGAAAGCCCGAGTTTAC-3′ R: 5′-GGACCTTTTTGGCTACTTGGC-3′
*Chalcone isomerase* (*CHI*)	F: 5′-TGGTGGCCTAGACAACGATGAGTT-3′ R: 5′-TCACACTCCCAACTTGGTTTCCCT-3′
*Flavonol synthase* (*FLS*)	F: 5′-TTAAAGGAAGGTCTCGGTGGCGAA-3′ R: 5′-TCATTGGTGACGATGAGTGCGAGT-3′
*Mevalonate kinase* (*MVK*)	F: 5′-TTATGTGTTGCGCTTTCAGC-3′ R: 5′-GAAGGCTTGCCATGAATGAT-3′
*Tannase enzyme* (*TA*)	F: 5′-GCAGTGCGTTGGAGCAATGGTGGGC-3′ R: 5′-CCCCGATACAAATCTGGGATAAGTG-3′

## Data Availability

The original contributions presented in the study are included in the article; further inquiries can be directed to the corresponding author.

## Supplementary Materials

The following supporting information can be downloaded at: https://www.mdpi.com/article/10.3390/plants14142093/s1, Figure S1. Characterization of Green-Synthesized Calcium Phosphate Nanoparticles (CaP-NPs) according to [56]. (a) UV-Visible absorption spectrum of CaP-NPs synthesized using Jania rubens extract, showing characteristic absorption peaks indicative of nanoparticle formation. (b) Transmission Electron Microscopy (TEM) image depicting the morphology and size distribution of CaP-NPs, demonstrating spherical particles with uniform size. (c) Fourier Transform Infrared (FTIR) spectrum of CaP-NPs, illustrating functional groups and chemical bonds confirming successful synthesis and surface capping by biomolecules from Jania rubens extract. Figure S2. HPLC Chromatograms of (A–D) Polyphenolic Profiles in Carob Callus Extracts under Different CaP-NP Treatments A, control, B, 25 mg/L C, 50 mg/L D, 75 mg/L CaP-NPs and (E) Polyphenol Standards. Figure S3. HPLC Chromatograms of (A) Tannin Standards (B–E) Tannin Profiles in Carob Callus Extracts under Different CaP-NP Treatments B, control, C, 25 mg/L D, 50 mg/L E, 75 mg/L CaP-NPs and. Table S1. Method validation parameters for HPLC analysis of secondary metabolites in carob callus cultures.
