# Supplementary material for: Biofortified Calcium Phosphate Nanoparticles Elicit Secondary Metabolite Production in Carob Callus via Biosynthetic Pathway Activation
Source: Plants (Basel). 2025 Jul 8;14(14):2093. doi: 10.3390/plants14142093 (PMC12300979; doi:10.3390/plants14142093)
Supplement: Supplementary file 1 [file plants-14-02093-s001.zip › plants-3687045-supplementary.pdf]

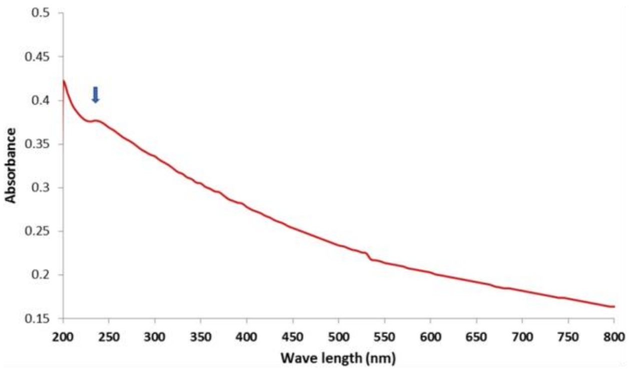

UV–Vis spectrum of biosynthesized CaP-NPs

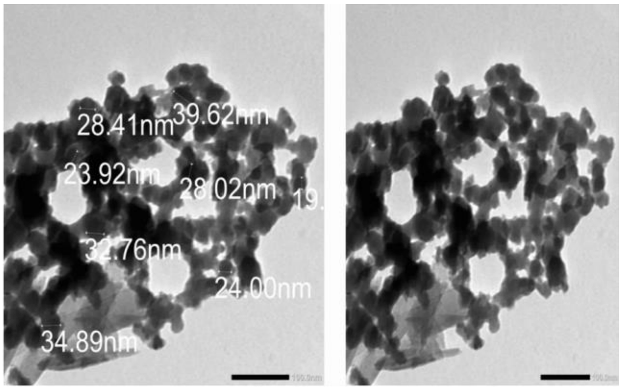

TEM image of biosynthesized CaP-NPs

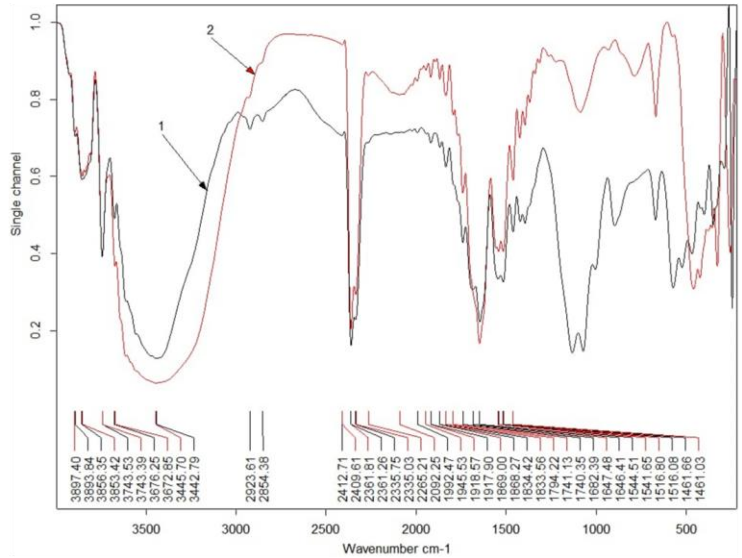

FT–IR spectra of (1) *Jania rubens* aqueous extract and (2) biosynthesized CaP-NPs

Figure 1 Supplementary Figure S1. Characterization of Green-Synthesized Calcium Phosphate Nanoparticles (CaP-NPs) according to [56]. (a) UV-Visible absorption spectrum of CaP-NPs synthesized using Jania rubens extract, showing characteristic absorption peaks indicative of nanoparticle formation.

(b) Transmission Electron Microscopy (TEM) image depicting the morphology and size distribution of CaP-NPs, demonstrating spherical particles with uniform size.

(c) Fourier Transform Infrared (FTIR) spectrum of CaP-NPs, illustrating functional groups and chemical bonds confirming successful synthesis and surface capping by biomolecules from Jania rubens extract.

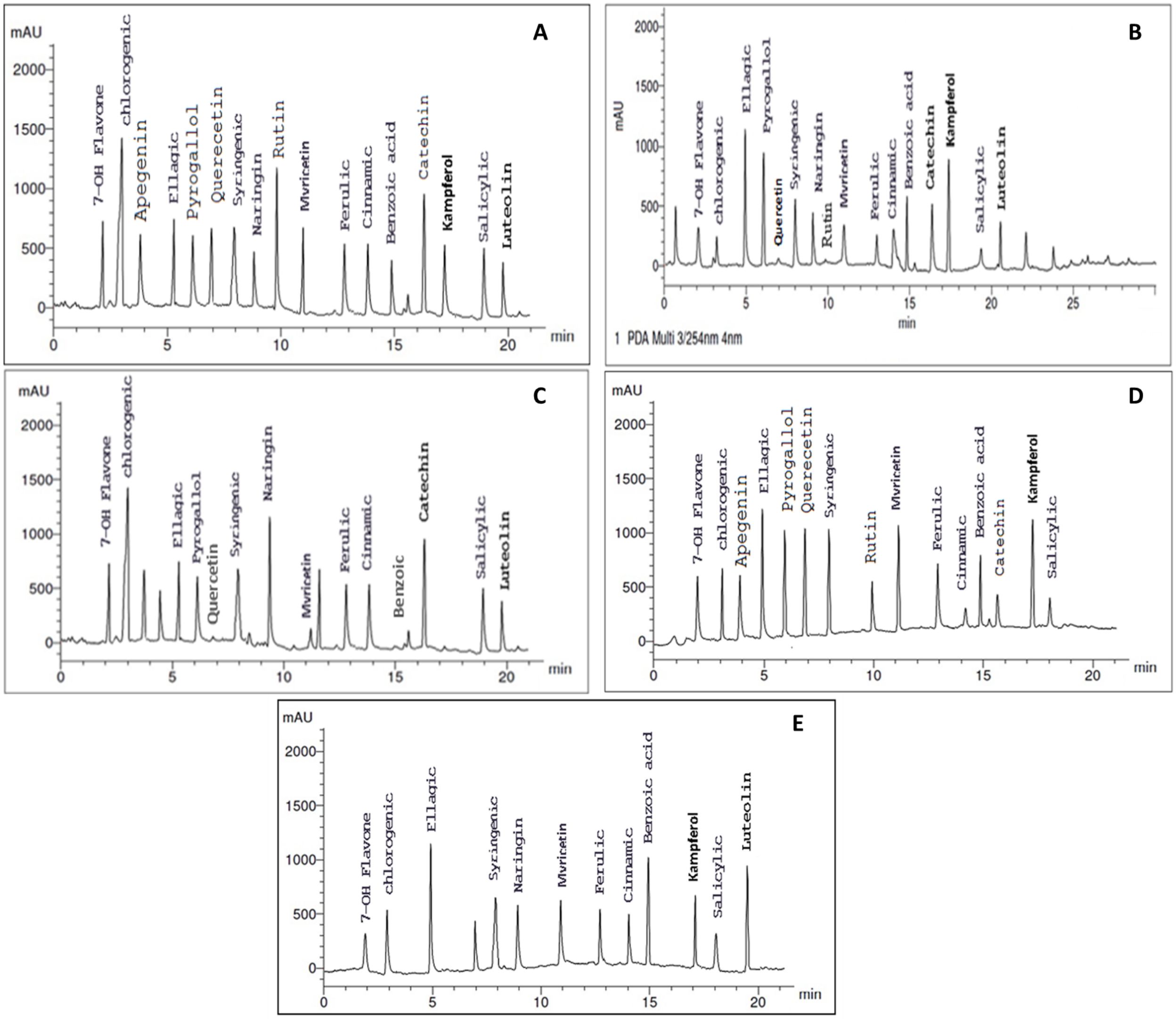

Figure 2 Supplementary Figure S2. HPLC Chromatograms of (A-D) Polyphenolic Profiles in Carob Callus Extracts under Different CaP-NP Treatments A, control, B,25 mg/L C,50 mg/L D,75 mg/L CaP-NPs and (E) Polyphenol Standards.

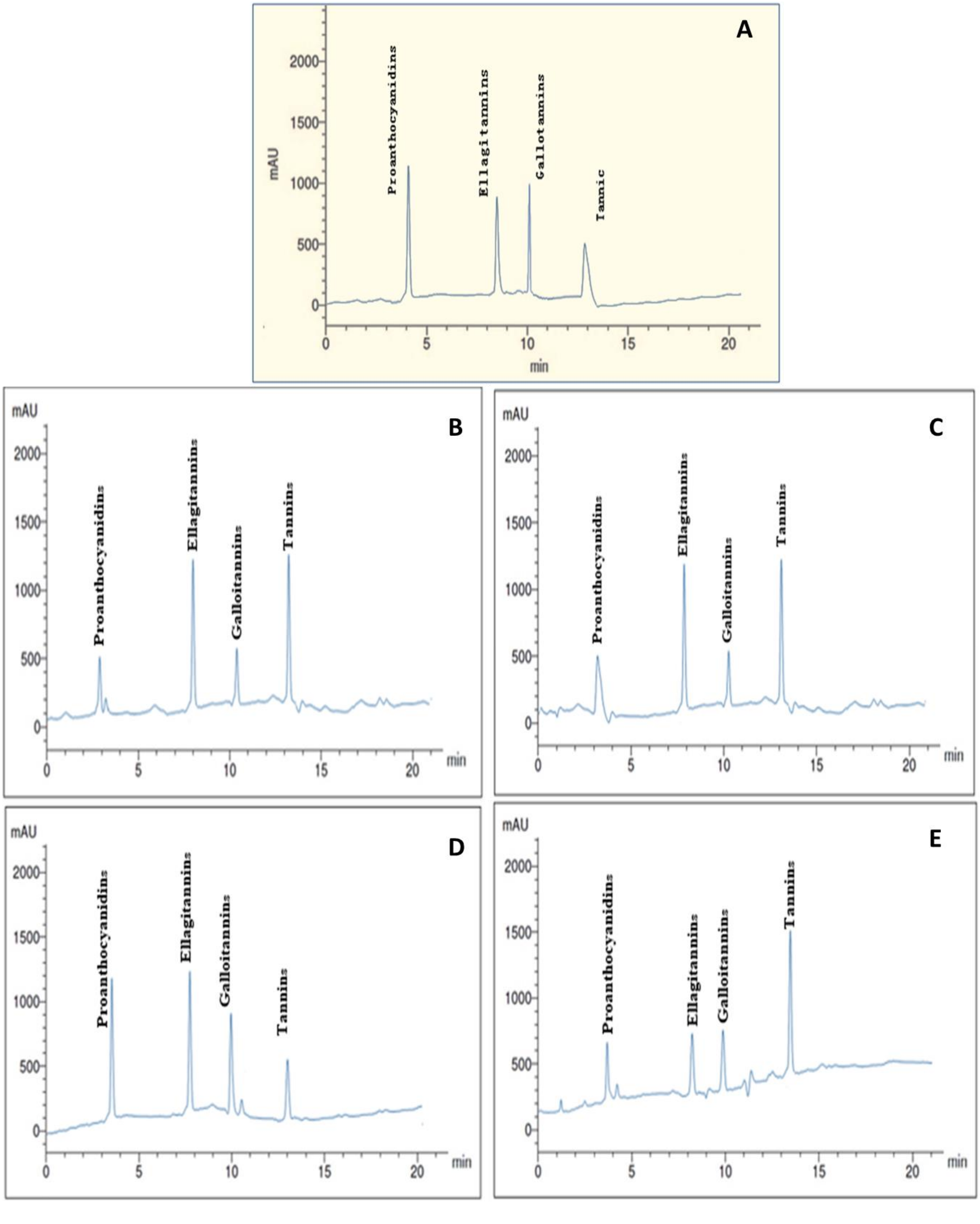

Figure 3 Supplementary Figure S3. HPLC Chromatograms of (A) Tannin Standards (B-E) Tannin Profiles in Carob Callus Extracts under Different CaP-NP Treatments B, control, C,25 mg/L D,50 mg/L E,75 mg/L CaP-NPs and.

Table 1 Supplementary Table S1. Method validation parameters for HPLC analysis of secondary metabolites in carob callus cultures.

Compound	Linearity Range (µg/mL)	Correlation Coefficient (R^2)	LOD (µg/mL)	LOQ (µg/mL)
Phenolics	5 – 100	> 0.995	0.02	0.066
Flavonoids	2 – 50	> 0.995	0.03	0.099
Tannins	1 – 40	> 0.995	0.045	0.15
Terpenoids	0.5 – 30	> 0.995	0.0901	0.297
